# Identification of SARS-CoV-2 biomarkers in saliva by transcriptomic and proteomics analysis

**DOI:** 10.1186/s12014-023-09417-w

**Published:** 2023-08-03

**Authors:** Lina M. Marin, George S. Katselis, Paulos Chumala, Stephen Sanche, Lucas Julseth, Erika Penz, Robert Skomro, Walter L. Siqueira

**Affiliations:** 1 College of Dentistry, University of Saskatchewan, Saskatoon, SK S7N 5E5 Canada; 2 Canadian Centre for Health and Safety in Agriculture, Department of Medicine, College of Medicine, University of Saskatchewan, Saskatoon, SK S7N 2Z4 Canada; 3 Division of Infectious Diseases, Department of Medicine, and Department of Pathology and Laboratory Medicine, College of Medicine, University of Saskatchewan, Saskatoon, SK S7N 0X8 Canada; 4 Division of Respirology, Critical Care and Sleep Medicine, Department of Medicine, College of Medicine, University of Saskatchewan, Saskatoon, SK S7N 0X8 Canada

**Keywords:** Saliva, SARS-CoV-2, Peptides, Proteins, Proteomics, Mass spectrometry, Transcriptomics, Real-time RT-PCR, Biomarkers

## Abstract

The detection of SARS-CoV-2 biomarkers by real time PCR (rRT-PCR) has shown that the sensitivity of the test is negatively affected by low viral loads and the severity of the disease. This limitation can be overcome by the use of more sensitive approaches such as mass spectrometry (MS), which has not been explored for the detection of SARS-CoV-2 proteins in saliva. Thus, this study aimed at assessing the translational applicability of mass spectrometry-based proteomics approaches to identify viral proteins in saliva from people diagnosed with COVID-19 within fourteen days after the initial diagnosis, and to compare its performance with rRT-PCR. After ethics approval, saliva samples were self-collected by 42 COVID-19 positive and 16 healthy individuals. Samples from people positive for COVID-19 were collected on average on the sixth day (± 4 days) after initial diagnosis. Viable viral particles in saliva were heat-inactivated followed by the extraction of total proteins and viral RNA. Proteins were digested and then subjected to tandem MS analysis (LC-QTOF-MS/MS) using a data-dependent MS/MS acquisition qualitative shotgun proteomics approach. The acquired spectra were queried against a combined SARS-CoV-2 and human database. The qualitative detection of SARS-CoV-2 specific RNA was done by rRT-PCR. SARS-CoV-2 proteins were identified in all COVID-19 samples (100%), while viral RNA was detected in only 24 out of 42 COVID-19 samples (57.1%). Seven out of 18 SARS-CoV-2 proteins were identified in saliva from COVID-19 positive individuals, from which the most frequent were replicase polyproteins 1ab (100%) and 1a (91.3%), and nucleocapsid (45.2%). Neither viral proteins nor RNA were detected in healthy individuals. Our mass spectrometry approach appears to be more sensitive than rRT-PCR for the detection of SARS-CoV-2 biomarkers in saliva collected from COVID-19 positive individuals up to 14 days after the initial diagnostic test. Based on the novel data presented here, our MS technology can be used as an effective diagnostic test of COVID-19 for initial diagnosis or follow-up of symptomatic cases, especially in patients with reduced viral load.

**Supplementary Information:**

The online version contains supplementary material available at 10.1186/s12014-023-09417-w.

## Introduction

COVID-19 pandemic, caused by SARS-CoV-2 coronavirus, has lasted for more than two years now. Public health measures imposed around the world to reduce the transmission rate of SARS-CoV-2 and to prevent health systems from being overwhelmed have not been sufficient to ease the pandemic. Mass-scale vaccination is a promising addition to public health efforts however, it has not been enough to eradicate the disease, due in part to the surge of more transmissible variants of the virus and the COVID-19 outbreaks especially in unvaccinated people [1]. Moreover, the real picture of the pandemic may have been underestimated due to the limited availability of testing, and protocols that often missed asymptomatic infected people.

Exploration of the SARS-CoV-2 viral genome [2] allowed the detection of SARS-CoV-2 to confirm suspected cases of COVID-19 by molecular biology assays [3]. Real-time RT-PCR (rRT-PCR), considered the gold standard method, is routinely used in samples obtained from naso/oropharyngeal swabs, sputum, or bronchoalveolar lavage fluid [3]. Apart from being an expensive technique, in most cases it requires a qualified healthcare worker to carry out the nasal swabbing process and a long waiting time to obtain results, making mass testing impossible [4, 5]. Moreover, the accuracy of rRT-PCR method can be jeopardized by inadequate sample collection, handling, and analysis [6] leading to false-negative results. To overcome these disadvantages, low-cost and rapid diagnostic methods based on the detection of SARS-CoV-2 antigens [7] or human antibodies against SARS-CoV-2 [8] have been developed. However, their inability to detect infected individuals at early stages or with low viral loads may also be a matter of concern [9, 10]. Therefore, there is an urgent need to develop simple and effective diagnostic platforms for COVID-19 that allow the large-scale screening of symptomatic and asymptomatic individuals, leading to the formulation of measures to control the transmission of COVID-19 and provide timely treatments at individual and population levels.

The detection of SARS-CoV-2 in the saliva of COVID-19 patients [11, 12] has provided a strong rationale to propose saliva as the most reliable tool to detect SARS-CoV-2 [13, 14]. In fact, saliva testing is attractive for disease diagnostics and monitoring of health conditions not only because of its multiple contributors, but also because its collection is non-invasive and painless [15, 16], and can be self-collected reducing the risk of transmission to health workers [14]. Despite advances in diagnostic salivary methods [5], to date the sensitivity of rRT-PCR in detecting SARS-CoV-2 biomarkers in saliva is negatively affected by low viral loads and the severity of the disease [17]. The limitations of the rRT-PCR method can be overcome by using more sensitive approaches such as mass spectrometry (MS), to detect viral biomarkers other than RNA, such as proteins and peptides. Identification of SARS-CoV-2 proteins in saliva by MS-based clinical proteomics has not been extensively explored [18], and the scarce proteomics research done has focused on the identification of viral proteins in naso/oropharyngeal swabs [19–26], gargle solutions [18, 27], and plasma [28]. The potential use of salivary proteomics for diagnostic purposes was previously demonstrated by our group [29] using a MS-based clinical proteomics approach to identify viral proteins and peptides in saliva from individuals with Zika fever, a disease also caused by an RNA virus.

Taking into account recent advances in clinical proteomics for the identification of multiple protein biomarkers for viral infections [29] and the shedding of SARS-CoV-2 through saliva [11, 12], it is expected that SARS-CoV-2 proteins can also be detected in saliva by means of MS-based proteomics. Thus, in this study we explored a new and uncharted area of COVID-19 diagnosis utilizing a proteomics approaches to identify SARS-CoV-2 proteins in saliva from people diagnosed with COVID-19 days after the initial diagnosis, when the viral load was expected to decrease [17], and compared its performance with the conventional rRT-PCR method (Table 1).

**Table 1:** Results from the proteomics and rRT-PCR analyses in saliva samples collected from COVID-19 positive and healthy individuals

Participant ID	Group	Proteomics								rRT-PCR		
		Non-structural proteins		Structural proteins			Accessory proteins		Proteomics diagnosis	Gene S (SARS-CoV-2)	Internal Control	rRT-PCR diagnosis
		R1A	R1AB	M	N	S	ORF3a	ORF9b				
1	COVID-19	x	x	–	–	–	–	x	Positive	–	x	Negative
2	COVID-19	x	x	x	–	x	–	x	Positive	–	x	Negative
3	COVID-19	x	x	x	–	–	x	–	Positive	–	x	Negative
4	COVID-19	x	x	–	x	x	x	–	Positive	x	x	Positive
5	COVID-19	x	x	–	x	x	x	–	Positive	–	x	Negative
6	COVID-19	x	x	–	x	x	x	–	Positive	x	x	Positive
7	COVID-19	–	x	–	–	–	x	x	Positive	x	x	Positive
8	COVID-19	x	x	–	x	-	x	–	Positive	x	x	Positive
9	COVID-19	x	x	–	x	x	–	x	Positive	–	x	Negative
10	COVID-19	x	x	x	x	x	–	–	Positive	x	x	Positive
11	COVID-19	–	x	–	x	x	–	–	Positive	–	x	Negative
12	COVID-19	x	x	–	x	–	–	–	Positive	x	x	Positive
13	COVID-19	x	x	–	x	x	–	–	Positive	x	x	Positive
14	COVID-19	x	x	–	x	–	–	–	Positive	–	x	Negative
15	COVID-19	x	x	x	x	x	–	–	Positive	–	x	Negative
16	COVID-19	x	x	–	x	–	–	–	Positive	–	x	Negative
17	COVID-19	x	x	–	x	–	–	–	Positive	x	x	Positive
18	COVID-19	x	x	x	–	x	–	–	Positive	x	x	Positive
19	COVID-19	x	x	-	–	–	–	–	Positive	x	x	Positive
20	COVID-19	x	x	–	x	–	–	–	Positive	x	x	Positive
21	COVID-19	x	x	–	–	–	–	–	Positive	x	x	Positive
22	COVID-19	x	x	–	x	–	–	–	Positive	–	x	Negative
23	COVID-19	x	x	x	–	–	–	x	Positive	–	x	Negative
24	COVID-19	x	x	–	–	–	–	–	Positive	–	x	Negative
25	COVID-19	x	x	–	x	–	–	–	Positive	x	x	Positive
26	COVID-19	x	x	–	–	–	–	–	Positive	–	x	Negative
27	COVID-19	x	x	–	–	–	–	x	Positive	–	x	Negative
28	COVID-19	–	x	–	–	–	–	–	Positive	x	x	Positive
29	COVID-19	x	x	–	–	–	–	x	Positive	x	x	Positive
30	COVID-19	x	x	x	x	x	–	x	Positive	x	x	Positive
31	COVID-19	-	x	–	–	–	–	–	Positive	x	x	Positive
32	COVID-19	x	x	–	–	–	–	–	Positive	–	x	Negative
33	COVID-19	x	x	–	–	–	–	–	Positive	x	x	Positive
34	COVID-19	x	x	–	–	–	–	–	Positive	x	x	Positive
35	COVID-19	x	x	–	–	–	–	–	Positive	–	x	Negative
36	COVID-19	x	x	–	–	–	–	–	Positive	x	x	Positive
37	COVID-19	x	x	–	–	–	–	–	Positive	x	x	Positive
38	COVID-19	x	x	–	–	–	–	–	Positive	x	x	Positive
39	COVID-19	x	x	–	–	–	–	–	Positive	x	x	Positive
40	COVID-19	x	x	–	–	–	–	–	Positive	x	x	Positive
41	COVID-19	x	x	–	–	–	–	–	Positive	–	x	Negative
42	COVID-19	x	x	–	–	–	–	–	Positive	–	x	Negative
C1	Healthy	–	–	–	–	–	–	–	Negative	–	x	Negative
C2	Healthy	–	–	–	–	–	–	–	Negative	–	x	Negative
C3	Healthy	–	–	–	–	–	–	–	Negative	–	x	Negative
C4	Healthy	–	–	–	–	–	–	–	Negative	–	x	Negative
C5	Healthy	–	–	–	–	–	–	–	Negative	–	x	Negative
C6	Healthy	–	–	–	–	–	–	–	Negative	–	x	Negative
C7	Healthy	–	–	–	–	–	–	–	Negative	–	x	Negative
C8	Healthy	–	–	–	–	–	–	–	Negative	–	x	Negative
C9	Healthy	–	–	–	–	–	–	–	Negative	–	x	Negative
C10	Healthy	–	–	–	–	–	–	–	Negative	–	x	Negative
C11	Healthy	–	–	–	–	–	–	–	Negative	–	x	Negative
C12	Healthy	–	–	–	–	–	–	–	Negative	–	x	Negative
C13	Healthy	–	–	–	–	–	–	–	Negative	–	x	Negative
C14	Healthy	–	–	–	–	–	–	–	Negative	–	x	Negative
C15	Healthy	–	–	–	–	–	–	–	Negative	–	x	Negative
C16	Healthy	–	–	–	–	–	–	–	Negative	–	x	Negative

*R1A* replicase polyprotein 1a (accession number: P0DTC1), *R1AB* replicase polyprotein 1ab (accession number: P0DTD1), *M* membrane (accession number: P0DTC5), *N* nucleocapsid (accession number: P0DTC9), *S* spike glycoprotein (accession number: P0DTC2); ORF3a (accession number: P0DTC3), ORF9b (accession number: P0DTD2); (x) identified; (–) not identified

## Materials and methods

### Selection of participants

This study was approved by the University of Saskatchewan Research Ethics Board (IRB#1911) and received Operational Approval from the Saskatchewan Health Authority (OA-UofS-1911). Male and female adults were invited to participate in this study and informed consent was obtained. The healthy control group was composed by two sets of samples. The first set consisted of samples collected from healthy individuals who were not experiencing COVID-19-related symptoms, had not travelled outside of Canada in the last 14 days, and had not had any contact with people diagnosed with COVID-19 (C1–C6, Table 1). The second set of samples consisted of saliva samples that were collected before the COVID-19 era and were stored in the saliva biobank at Salivary Proteomics Research Laboratory, University of Saskatchewan (C7–C16, Table 1), following the protocol of saliva collection described elsewhere [29]. Individuals assigned to the COVID-19 positive group were eligible to participate in this study only if received a positive result from the rRT-PCR test done by the Saskatchewan Health Authority (SHA) through a nasopharyngeal swab (NPS). COVID-19 positive individuals residing in Saskatoon and surrounding area were informed by staff members of SHA about this study and were instructed to contact the research team if they were interested in participating in the study. Those interested in participating were screened by a member of the research team for exclusion criteria that included: any history of chronic lung or heart disease (COPD, asthma, heart failure); any symptoms of COVID-19 respiratory syndrome without a confirmatory rRT-PCR test performed by the SHA; use of prescription medications, other than antibiotics at the time of enrollment or in the last three months; and presence of physical or mental illness with motor and/or cognitive impairment(s) that, in the opinion of the researcher, could interfere with compliance or outcomes.

### Saliva collection and processing

The first set of saliva samples from healthy volunteers (C1–C6, Table 1) were collected at Salivary Proteomics Research Laboratory, University of Saskatchewan. Saliva samples from people positive for COVID-19 were collected at each participants’ home within 14 days after receiving a positive test result from the COVID-19 test done by SHA. Stimulated whole saliva was self-collected using a collection kit (SimplOFy™, Oasis Diagnostics® Corporation, USA) without the addition of DNA stabilizers, a protocol that did not affect the stability of RNA or proteins (Additional file 1: Materials and Methods, and Figures S1 and S2). Saliva was stimulated by chewing a piece of parafilm [30]. Immediately after collection, saliva samples were sealed, labeled, and placed on ice to be transported to the Salivary Proteomics Research Laboratory, following all guidelines related to transportation of biohazardous materials. Upon arrival to the laboratory, viable SARS-CoV-2 particles in saliva were inactivated at 60 °C for 30 min [31], a protocol that did not affect protein stability (Additional file 1: Figure S2). Heat-inactivation also reduces the biological risk of infection, allowing samples to be handled in a Biosafety Level 2 laboratory [24]. To maintain consistency in sample handling and processing, saliva from healthy individuals was also heat-treated. After heat-inactivation, 1 mL of whole saliva was transferred to a centrifuge tube, centrifuged at 14,000 × *g* for 20 min at 4 °C to separate the pellet from whole saliva supernatant (WSS) [32]. WSS was transferred to a new centrifuge tube and kept on ice for further protein and RNA extraction.

### Protein extraction from WSS

WSS proteins were purified by ice-cold acetone precipitation. Extracted proteins were reconstituted in 100 mM ammonium bicarbonate and mixed to obtain a whole saliva protein extract. Protein concentration in the whole saliva protein extract was measured using the BCA assay kit (Pierce™). The equivalent of 40 µg of protein from each sample was dried in SpeedVac (Labconco, USA) and stored at − 80 °C.

### MS-based proteomics workflow

Dried proteins from the whole saliva protein extract were reduced, alkylated, and digested with trypsin in-solution following an in-house developed protocol [33]. After digestion, all peptide samples were reconstituted in MS grade water:acetonitrile:formic acid (97:3:0.1 v/v) to a final concentration of 0.5 µg/µL. A 15 µL aliquot of each sample was transferred to a MS vial for liquid chromatography-tandem mass spectrometry (LC–MS/MS) analysis. Mass spectral analyses were performed on an Agilent 6550 iFunnel quadrupole time-of-flight (QTOF) mass spectrometer equipped with an Agilent 1260 series liquid chromatography instrument and an Agilent Chip Cube LC–MS interface (Agilent Technologies Canada Ltd., Mississauga, ON, CA). Each sample was analysed in triplicate by injecting 2 µg of tryptic peptides per run. A blank solution (0.1% formic acids and 90% acetonitrile in water) was run between samples to avoid carry-over between analytical runs. Chromatographic peptide separation was accomplished using a high-capacity high performance LC-Chip II: G4240-62,030 Polaris-HR-Chip_3C18 consisting of a 360 nL enrichment column and a 75 µm × 150 mm analytical column, both packed with Polaris C18-A, 180 Å, 3 µm stationary phase. Samples were loaded onto the enrichment column with solvent A (0.1% formic acid in water) at a flow rate of 2.0 µL/min. Samples loaded to enrichment column were transferred onto the analytical column, and peptides were separated with a linear gradient solvent system of solvent A and solvent B (0.1% formic acid in acetonitrile), as follows: 3–25% B for 105 min, 25–40% B for 15 min, 40–90% B for 5 min, where it remained there for 5 min and then returned to the initial conditions, where the column was equilibrated for 5 min, at a flow rate of 0.3 µL/min. Positive-ion electrospray MS data were acquired using a capillary voltage set at 1900 V, the ion fragmentor set at 360 V, and the drying/collision gas (nitrogen) set at 225 °C with a flow rate of 12.0 L/min. Spectral results were collected over a mass range of 250–1700 mass/charge (m/z) at a scan rate of 8 spectra/sec. Tandem mass spectrometry (MS/MS) data were collected over a range of 100–1700 m/z and a set isolation width of 1.3 atomic mass units. The top 20 most intense precursor ions for each MS scan were selected for MS/MS with a 0.25 min active exclusion.

### Bioinformatics analyses

Tandem mass spectra were extracted from raw data, converted to a mass/charge data format using Agilent MassHunter Qualitative Analysis Software (Agilent Technologies Canada Ltd., Mississauga, ON, CA), and queried against a combined SARS-CoV-2 and human database (UniProt, both downloaded on June 25, 2021) consisting of 20,403 reviewed proteins (SwissProt), using Spectrum Mill (Agilent Technologies Canada Ltd., Mississauga, ON, CA) as the database search engine. Search parameters included a fragment mass error of 50 parts per million (ppm), a parent mass error of 20 ppm, trypsin cleavage specificity (two missed cleavages per peptide), and carbamidomethylation as a fixed modification of cysteine. Oxidized methionine, carbamylated lysine, pyroglutamic acid, deamidated asparagine, phosphorylated serine, threonine, and tyrosine and acetyl lysine were set as variable modifications. Data were also searched using semi-trypsin non-specific C- and N-terminus to increase protein identification. Spectrum Mill results were validated at peptide and protein levels (1% false discovery rate) and by manually inspecting the MS/MS spectra to confirm the identity of signature b- and y-fragment ions. The sequence from the tryptic peptides derived from replicase polyprotein isoform 1a and isoform 1ab were queried against the non-redundant protein sequences database using the blastp (protein–protein Basic Local Alignment Search Tool) algorithm [34]. The blastp approach allowed the identification of the non-structural proteins (nsps) in saliva.

### RNA extraction from WSS and rRT-PCR

Concurrently with the proteomics analysis, saliva samples were tested using rRT-PCR. rRT-PCR tests were done in all saliva samples to compare the sensitivity and specificity of this test with our proteomics approach at the time of saliva sample collection, which differs from the time of initial COVID-19 diagnosis. For this, total RNA was extracted from WSS using QIAmp Viral RNA Mini Kit (Qiagen). Extracted RNA was used for the qualitative detection of SARS-CoV-2 (target S gene coding for the membrane fusion subunit, domain 2, of the spike protein) specific RNA in saliva with RealStar® SARS-CoV-2 RT-PCR Kit 1.0 reagent system (Altona Diagnostics GmbH), based on rRT-PCR technology, using a CFX96™ Touch Real-Time PCR Detection System (Bio-Rad). Both RNA extraction and rRT-PCR were done according to the manufacturer's instructions. Samples were considered positive for COVID-19 by rRT-PCR analysis if having Cq values ≤ 38.

## Results

Stimulated whole saliva was obtained from forty-two COVID-19 positive individuals, 16 female and 26 male; and from sixteen healthy individuals, 8 female and 8 male. The mean age of the individuals in the COVID-19 group was of 40.8 (± 13.4) and 39.9 (± 17.3) years for female and male participants, respectively. The mean age in the healthy control group was of 37.9 (± 13.0) for female and 34.1 (± 12.1) for male participants. On average, saliva samples from COVID-19 positive individuals were collected on the 6^th^ day (± 4 days) after the confirmatory NPS by rRT-PCR done by Saskatchewan Health Authority (SHA).

Our MS approach allowed the identification of 1369 proteins from human origin and 7 SARS-CoV-2 proteins in saliva of COVID-19 positive individuals (Table 2). From the identified SARS-CoV-2 proteins, three correspond to the virus structural proteins membrane (accession number: P0DTC5), nucleocapsid (accession number: P0DTC9), and spike glycoprotein (accession number: P0DTC2); two to the non-structural proteins replicase polyprotein isoform 1a (accession number: P0DTC1) and isoform 1ab (accession number: P0DTD1); and two to the accessory proteins ORF3a (accession number: P0DTC3) and ORF9b (accession number: P0DTD2) (Table 2). The most frequent viral proteins identified among people assigned to the COVID-19 group were replicase polyproteins 1ab (100%) and 1a (91.3%), followed by nucleocapsid (45.2%) (Table 2).

**Table 2:** List of SARS-CoV-2 proteins identified in individuals positive for COVID-19 and frequency of identification

Accession number	Protein name	Frequency	
		n	%
P0DTC1	Replicase polyprotein 1a	38	91.3
P0DTD1	Replicase polyprotein 1ab	42	100.0
P0DTC5	Membrane protein	7	16.7
P0DTC9	Nucleocapsid	19	45.2
P0DTC2	Spike glycoprotein	11	26.2
P0DTC3	ORF3a protein	6	14.3
P0DTD2	ORF9b protein	8	19.0

The data obtained from the proteomics analysis were compared with rRT-PCR analysis, both done on saliva samples. The results showed that SARS-CoV-2 specific RNA was detected in only 24 out of 42 people positive for COVID-19, while viral proteins were identified in all samples analyzed. Neither SARS-CoV-2 proteins nor RNA were detected in saliva samples from healthy individuals (Tables 1 and 3), while 1362 proteins from human origin were identified in these samples by MS. The Cq values obtained for the detection of SARS-CoV-2 (target S gene) specific RNA are provided in Table 4.

**Table 3:** Sensitivity and specificity of proteomics and rRT-PCR analyses done in saliva samples collected from COVID-19 positive and healthy individuals

		COVID-19 status				COVID-19 status	
		Positive	Healthy			Positive	Healthy
Proteomics analysis	Positive	n = 42	n = 0	rRT-PCR analysis	Positive	n = 24	n = 0
	Negative	n = 0	n = 16		Negative	n = 18	n = 16
Total		n = 42	n = 16	Total		n = 42	n = 16
Sensitivity and specificity		Sensitivity 1.0 (100%)	Specificity 1.0 (100%)	Sensitivity and Specificity		Sensitivity 0.57 (57.1%)	Specificity 1.0 (100%)

**Table 4:** Cq values obtained during the detection of SARS-CoV-2 (target S gene) specific RNA by rRT-PCR in saliva samples collected from COVID-19 positive and healthy individuals, respectively

Participant ID	Group	Gene S (SARS-CoV-2)	rRT-PCR diagnosis
		Cq	
1	COVID-19	0	Negative
2	COVID-19	0	Negative
3	COVID-19	0	Negative
4	COVID-19	32.2	Positive
5	COVID-19	0	Negative
6	COVID-19	30.9	Positive
7	COVID-19	30.6	Positive
8	COVID-19	28.0	Positive
9	COVID-19	0	Negative
10	COVID-19	23.4	Positive
11	COVID-19	0	Negative
12	COVID-19	25.1	Positive
13	COVID-19	29.5	Positive
14	COVID-19	0	Negative
15	COVID-19	0	Negative
16	COVID-19	0	Negative
17	COVID-19	34.3	Positive
18	COVID-19	28.8	Positive
19	COVID-19	35.5	Positive
20	COVID-19	35.9	Positive
21	COVID-19	31.2	Positive
22	COVID-19	0	Negative
23	COVID-19	0	Negative
24	COVID-19	0	Negative
25	COVID-19	35.0	Positive
26	COVID-19	0	Negative
27	COVID-19	0	Negative
28	COVID-19	28.1	Positive
29	COVID-19	25.9	Positive
30	COVID-19	34.3	Positive
31	COVID-19	24.5	Positive
32	COVID-19	0	Negative
33	COVID-19	33.4	Positive
34	COVID-19	24.2	Positive
35	COVID-19	0	Negative
36	COVID-19	30.7	Positive
37	COVID-19	26.1	Positive
38	COVID-19	36.0	Positive
39	COVID-19	34.7	Positive
40	COVID-19	32.0	Positive
41	COVID-19	0	Negative
42	COVID-19	0	Negative
C1	Healthy	0	Negative
C2	Healthy	0	Negative
C3	Healthy	0	Negative
C4	Healthy	0	Negative
C5	Healthy	0	Negative
C6	Healthy	0	Negative
C7	Healthy	0	Negative
C8	Healthy	0	Negative
C9	Healthy	0	Negative
C10	Healthy	0	Negative
C11	Healthy	0	Negative
C12	Healthy	0	Negative
C13	Healthy	0	Negative
C14	Healthy	0	Negative
C15	Healthy	0	Negative
C16	Healthy	0	Negative

**Fig. 1:**
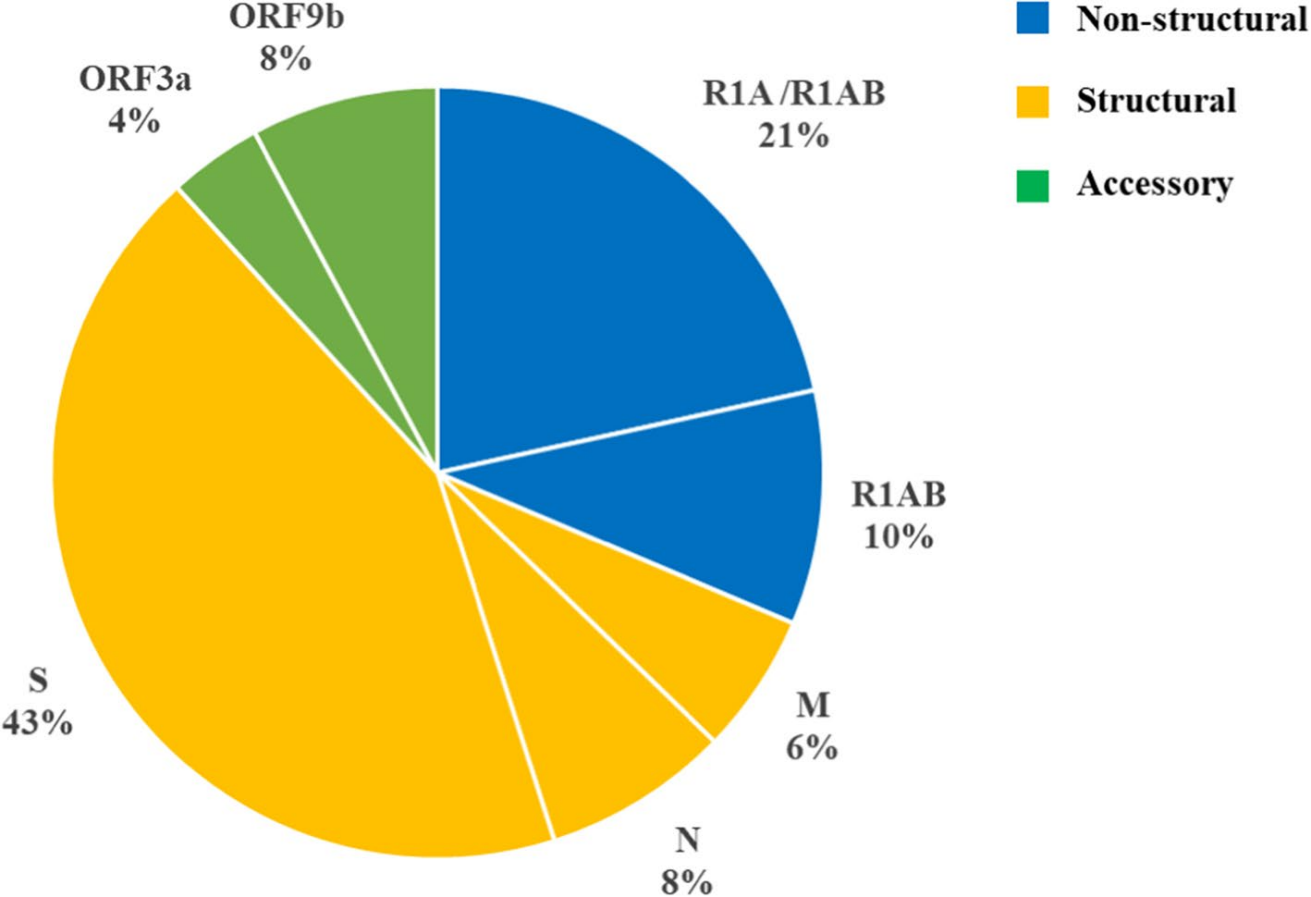
Distribution of tryptic peptides identified in saliva samples collected from COVID-19 positive individuals. *R1A* replicase polyprotein 1a (accession number: P0DTC1), *R1AB* replicase polyprotein 1ab (accession number: P0DTD1), *M* membrane (accession number: P0DTC5), *N* nucleocapsid (accession number: P0DTC9), *S* spike glycoprotein (accession number: P0DTC2); ORF3a (accession number: P0DTC3); ORF9b (accession number: P0DTD2)

**Fig. 2:**
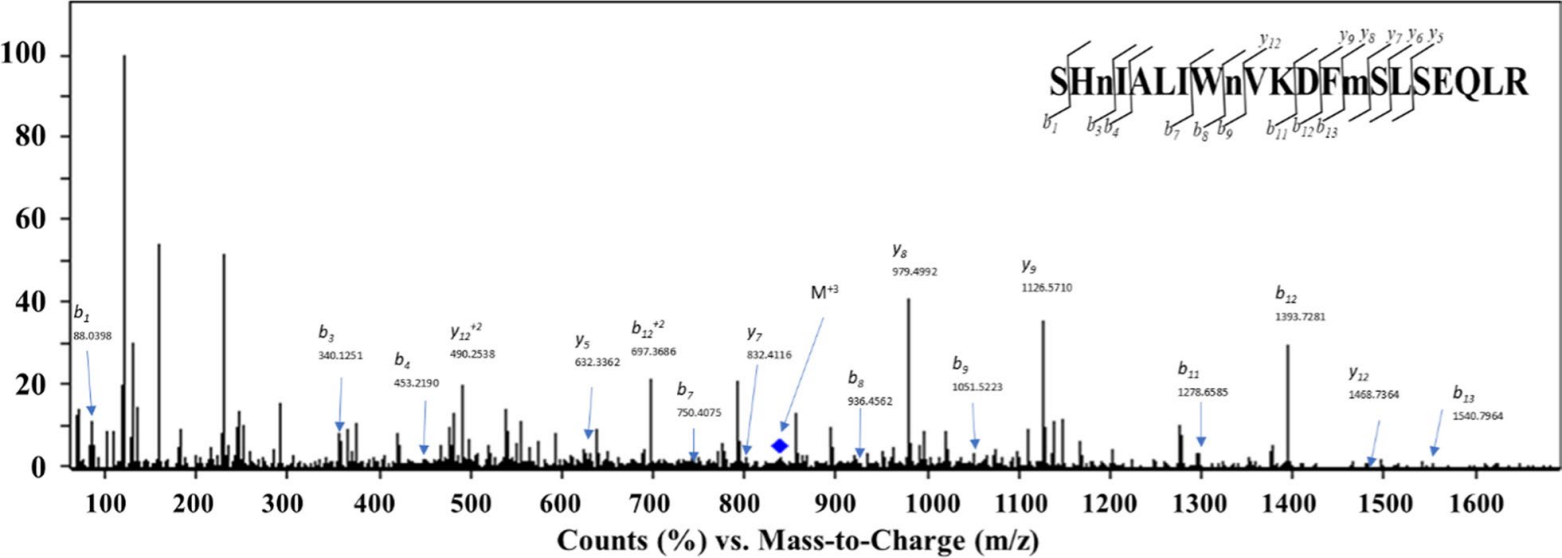
MS/MS spectra and amino acid sequence analysis of the tryptic peptide most frequently identified in saliva from COVID-19 positive individuals. Tryptic peptide derived from both isoforms of SARS-CoV-2 replicase polyproteins 1a (accession number: P0DTC1) and 1ab (accession number: P0DTD1). The amino acid sequence of the peptide is – SHnIALIWnVKDFmSLSEQLR –. Triply charged precursor ion indicated by the diamond shape; m/z = 840.438, z = 3. B). Matching b- and y- ion series are indicated in the upper right of each figure

Regarding the tryptic peptides identified, the most abundant were those originated from spike glycoprotein (43%) and replicase polyproteins 1a/1ab (31%) (Fig. 1), while the most frequent tryptic peptide identified among COVID-19 positive individuals was the one originated from both replicase polyprotein isoforms [(K)-SHnIALIWnVKDFmSLSEQLR-(K), 73.8%] (Fig. 2 and Table 5). In total, 51 tryptic peptides originated from SARS-CoV-2 proteins were identified in saliva from COVID-19 positive individuals.

**Table 5:** Fragment b- and y-ions attributed to the tryptic peptide SHnIALIWnVKDFmSLSEQLR as identified by tandem MS. The table also includes complementary fragment ions that aided in the assignment of the peptide

	Calculated mass	Measured mass	Fragment b-ions	Calculated mass	Measured mass
*Fragment y-ions*					
y1^+^	175.1190	ND	b1^+^	88.0393	88.0398
y2^+^	288.1554	ND	b2^+^	225.0982	ND
y3^+^	416.2616	ND	b3^+^	340.1252	340.1251
y4^+^	545.3042	ND	b4^+^	453.2092	453.2190
y5^+^	632.3362	632.3167	b5^+^	524.2463	ND
y6^+^	745.4203	745.3849	b6^+^	637.3304	ND
y7^+^	832.4523	832.4116	b7^+^	750.4145	750.4075
y8^+^	979.4877	979.4992	b8^+^	936.4938	936.4562
y9^+^	1126.5561	1126.5710	b9^+^	1051.5207	1051.5223
y10^+^	1241.5831	ND	b10^+^	1150.5891	ND
y11^+^	1369.678	ND	b11^+^	1278.6841	1278.6585
y12^+^	1468.7464	1468.7364	b12^+^	1393.711	1393.7281
y13^+^	1583.7734	ND	b13^+^	1540.7795	1540.7964
*Complementary ions*					
LS-H_2_O	183.1128	183.1166	b11^+2^	639.8457	639.8275
nI	229.1183	229.1239	b12^+2^	697.3592	697.3686
y12^+2^	490.2537	490.2538	y9^+^-NH_3_	1109.5296	1109.5421
y9^+^-NH_3_	555.2684	555.2804	LIWnVKDFm-NH_3_	1147.5492	1147.6083
y8^+^-NH_3_	962.4612	962.4727	KDFmSLSEQL-28	1167.5714	1167.6321
y11^+^-NH_3_	1352.6515	1352.6648	WnVKDFmS-28	997.4448	997.5106
EQ-28	230.1245	230.1247	VKDFmSLSEQL-H_2_O	1276.6242	1276.6532
EQ-NH_3_	241.0819	241.0861	VKDF-NH_3_	473.2395	473.2306
(y8-NH_3_)^+2^	481.7342	481.7397	KD	244.1292	244.1135

*ND* not detected

Considering that both isoforms of SARS-CoV-2 replicase polyprotein are cleaved into several non-structural proteins (nsps) [35], we used blastp algorithm to determine which nsps were detected in saliva of COVID-19 positive individuals. Our approach allowed identification of 5 out of 10 nsps common to both isoforms of replicase polyprotein (1a/1ab), and 4 out of 7 nsps specific to isoform 1ab (Table 6).

**Table 6:** List of replicase polyproteins 1a and 1ab tryptic peptides identified in saliva samples collected from COVID-19 positive individuals grouped by the polyprotein fragment to which they belong and their functions

Protein name	Peptide sequence	Polyprotein fragment name	Polyprotein fragment function
R1A/R1AB	LTDNVYIK	Nsp-3	Papain-like protease
	RVLNVVCK		
	TFYVLPNDDTLR		
	TTEVVGDIILKPANNSLK		
	IALKGGK	Nsp-4	NS
	IVNNWLKQLIK		
	IVQLSEISMDNSPNLAWPLIVTALR		
	VIGHSMQNCVLKLK	Nsp-5	3C-like proteinase
	KSLNVAK	Nsp-8	NS
	SHnIALIWnVKDFmSLSEQLR		
	WARFPK	Nsp-9	NS
R1AB	LKLFDR	Nsp-12	RNA-directed RNA polymerase
	EFLTRNPAWR	Nsp-13	Helicase
	VSAKPPPGDQFK	Nsp-14	Proofreading exonuclease
	NLQEFKPR	Nsp-15	Uridylate-specific endoribonuclease
	QASLNGVTLIGEAVK		

*R1A* replicase polyprotein 1a (accession number: P0DTC1), *R1AB* replicase polyprotein 1ab (accession number: P0DTD1), *Nsp* Non-structural protein, *NS* Non-specific

## Discussion

In this study, we characterized the SARS-CoV-2 proteome in saliva from individuals diagnosed with COVID-19, exploring a novel area of COVID-19 diagnosis through the combined use of saliva and MS-based proteomics. Based on the exciting data presented herein, we proved the concept that SARS-CoV-2 proteins can be detected in saliva from COVID-19 positive individuals up to 14 days after the initial diagnosis, when viral loads are expected to decrease.

Our protocol allowed us to identify 7 out of 17 SARS-CoV-2 proteins in saliva of COVID-19 positive individuals (Table 2), covering 41% of viral proteome (75% structural proteins, 100% non-structural proteins, 18.2% accessory proteins). SARS-CoV-2 proteins present in saliva might originate from free viral particles produced in the lower and upper respiratory tracts, salivary glands, and surrounding tissues, or delivered from the blood to the oral cavity via the gingival crevicular fluid [14]. Moreover, the identified structural viral proteins might be derived from free virions in saliva, while non-structural proteins and accessory proteins might represent active viral replication or release from lysed infected cells [29]. Based on our results, we speculate that our MS-based proteomics approach may allow for the identification of COVID-19 cases at different stages of the disease, since all three kinds of viral proteins (structural, non-structural, and accessory) were detected in the saliva samples analysed (Table 1).

All 51 tryptic peptides identified in the 42 COVID-19 positive individuals were unique to SARS-CoV-2 (Fig. 1), one of which was consistently found in most of the samples: (K)- SHnIALIWnVKDFmSLSEQLR-(K), from replicase polyproteins 1a and 1ab (73.8%), (Fig. 2). Although there are no reports on the identification of replicase polyproteins 1a/1ab tryptic peptides in human biofluids, our results suggest that these proteins can be considered as target compounds for diagnosing COVID-19 by MS-based proteomics. Apart from (K)- SHnIALIWnVKDFmSLSEQLR-(K) being the tryptic peptide most frequently identified, we were also able to detect most nsps resulting from the cleavage of both replicase polyprotein isoforms, 1a and 1ab, in saliva from all COVID-19 positive samples (Table S2). The nsps detected participate in essential processes of COVID-19 pathogenesis such as viral RNA replication and transcription, and immune evasion [35], confirming the applicability of our proteomics approach to detect COVID-19 cases at different stages of the disease.

Although saliva-based sampling for SARS-CoV-2 detection via rRT-PCR has shown to be reliable for the initial diagnosis of COVID-19 [4], our proteomics-based approach demonstrated better sensitivity than rRT-PCR to detect viral biomarkers in saliva, since SARS-CoV-2 proteins were identified in all samples, even in those which were rRT-PCR negative at the time of sample collection (Table 3). Since saliva samples used in this study were collected approximately six days after the confirmatory NPS test by rRT-PCR done by SHA, our results are potentially explained by the reduced viral load at the time of sample collection [5, 17], and by the higher rate of RNA degradation compared to that of the proteins [29], confirming the limitations of rRT-PCR test for the detection of SARS-CoV-2 biomarkers in saliva days after symptoms onset [17]. The increased lifetime of SARS-CoV-2 proteins in saliva might be due to protein–protein interactions, since viral proteins self-associate forming dimers or oligomers, or interact with other proteins from human or viral origin [36]. These physiological interactions may have protected the viral proteins from the proteolytic degradation that occurs in the oral cavity by salivary proteases from human and bacterial origin [37], allowing them to remain for longer periods of time as intact proteins in saliva. These mechanisms enable the detection of SARS-CoV-2 proteins by state-of-the-art proteomics approaches and help to explain the higher sensitivity obtained with this method compared to that of rRT-PCR (Table 3). Considering that there is a direct relationship among viral load, disease severity, and the detection of SARS-CoV specific RNA in saliva [17], further studies should be done to compare the sensitivity and specificity of our MS-based clinical proteomics approach with rRT-PCR in the detection of COVID-19 cases in saliva samples collected at the time of initial diagnosis, when the viral load is highest [17].

In addition to being highly sensitive, a diagnostic method must provide results in a timely manner, facilitating the early detection of COVID-19 cases or their accurate diagnosis [5]. In this way, sample analysis by MS may be expedited if the method is adapted to identify target tryptic viral peptides [20, 23, 24, 38] or to identify naturally occurring SARS-CoV-2 peptides in saliva. The main advantage of the latter approach is that the sample can be analysed directly, eliminating the preparation process required for the bottom-up proteomics strategy used in this study [29]. This method allows the identification of natively cleaved SARS-CoV-2 peptides in saliva, a methodology previously reported by our group that proved useful in the identification of Zika virus peptides in saliva [29]. In this regard, further studies will test the applicability of our high-throughput MS-based peptidomics method [29] for the identification of native SARS-CoV-2 peptides in saliva of COVID-19 positive individuals. The SARS-CoV-2 native cleaved peptides identified in saliva will facilitate the development of COVID-19 point-of-care diagnostic assays, which could easily be scaled up in numerous locations, adding much-needed testing capacity.

Regarding the methodological aspects, the protocol of saliva self-collection used in this study demonstrates that this biofluid can be self-collected anywhere using the non-invasive technique reported herein. In terms of sample stability, the use of saliva for proteomics analyses demonstrated to be ideal for COVID-19 diagnosis. As mentioned in the methods section, saliva samples were kept on ice immediately after collection, a protocol that prevents proteolytic degradation without interfering with the chemistry of the proteome [39]. Moreover, the method of saliva collection and processing used in this study did not lead to RNA degradation, as demonstrated by the similar rRT-PCR results of saliva samples collected using three different methods (Additional file 1: Figure S1). Although requiring further confirmation with saliva samples collected at a large cohort and comparison with the gold-standard rRT-PCR naso/oropharyngeal swab method at the time of the initial diagnosis test [3], here we demonstrated the applicability of our MS-based proteomics technique for the identification of SARS-CoV-2 proteins in saliva from COVID-19 positive individuals. Our findings reinforce the advantages of using MS over rRT-PCR for the detection and follow-up of COVID-19 cases [23, 40], especially in cases with reduced viral load that cannot be detected by rRT-PCR [17].

### Supplementary Information

**Additional file 1: **rRT-PCR amplification curves of stimulated whole saliva samples self-collected by COVID-19 positive individuals using three different collection methods: standard, ice, and RNAlater (**Figure S1**); SDS-PAGE of stimulated whole saliva samples before and after heat treatment at 60 °C for 30 min (**Figure S2**).

## Data Availability

The datasets used and/or analysed during the current study are available from the corresponding author on reasonable request.

## References

[1] Goldman E (2021). How the unvaccinated threaten the vaccinated for COVID-19: a Darwinian perspective. Proc Natl Acad Sci U S A.

[2] Wu F, Zhao S, Yu B, Chen YM, Wang W, Song ZG (2020). A new coronavirus associated with human respiratory disease in China. Nature.

[3] Corman VM, Landt O, Kaiser M, Molenkamp R, Meijer A, Chu DK (2020). Detection of 2019 novel coronavirus (2019-nCoV) by real-time RT-PCR. Euro Surveill.

[4] Bastos ML, Perlman-Arrow S, Menzies D, Campbell JR (2021). The sensitivity and costs of testing for SARS-CoV-2 infection with saliva versus nasopharyngeal swabs: a systematic review and meta-analysis. Ann Intern Med.

[5] Azzi L, Maurino V, Baj A, Dani M, d’Aiuto A, Fasano M (2020). Diagnostic salivary tests for SARS-CoV-2. J Dent Res.

[6] Lippi G, Simundic AM, Plebani M (2020). Potential preanalytical and analytical vulnerabilities in the laboratory diagnosis of coronavirus disease 2019 (COVID-19). Clin Chem Lab Med.

[7] Stadlbauer D, Amanat F, Chromikova V, Jiang K, Strohmeier S, Arunkumar GA (2020). SARS-CoV-2 seroconversion in humans: a detailed protocol for a serological assay, antigen production, and test setup. Curr Protoc Microbiol.

[8] Li Z, Yi Y, Luo X, Xiong N, Liu Y, Li S (2020). Development and clinical application of a rapid IgM-IgG combined antibody test for SARS-CoV-2 infection diagnosis. J Med Virol.

[9] Tang YW, Schmitz JE, Persing DH, Stratton CW (2020). The laboratory diagnosis of COVID-19 infection: current issues and challenges. J Clin Microbiol.

[10] To KK, Tsang OT, Leung WS, Tam AR, Wu TC, Lung DC (2020). Temporal profiles of viral load in posterior oropharyngeal saliva samples and serum antibody responses during infection by SARS-CoV-2: an observational cohort study. Lancet Infect Dis.

[11] Zheng S, Fan J, Yu F, Feng B, Lou B, Zou Q (2020). Viral load dynamics and disease severity in patients infected with SARS-CoV-2 in Zhejiang province, China, January-March 2020: retrospective cohort study. BMJ.

[12] To KK, Tsang OT, Chik-Yan Yip C, Chan KH, Wu TC, Chan JMC (2020). Consistent detection of 2019 novel coronavirus in saliva. Clin Infect Dis.

[13] Moreira VM, Mascarenhas P, Machado V, Botelho J, Mendes JJ, Taveira N (2021). Diagnosis of SARS-Cov-2 infection by RT-PCR using specimens other than naso- and oropharyngeal swabs: a systematic review and meta-analysis. Diagnostics (Basel)..

[14] Sabino-Silva R, Jardim ACG, Siqueira WL (2020). Coronavirus COVID-19 impacts to dentistry and potential salivary diagnosis. Clin Oral Investig.

[15] Wong DT (2006). Salivary diagnostics. J Calif Dent Assoc.

[16] Siqueira WL, Dawes C (2011). The salivary proteome: challenges and perspectives. Proteomics Clin Appl.

[17] Khiabani K, Amirzade-Iranaq MH (2021). Are saliva and deep throat sputum as reliable as common respiratory specimens for SARS-CoV-2 detection? A systematic review and meta-analysis. Am J Infect Control.

[18] Kipping M, Tänzler D, Sinz A (2021). A rapid and reliable liquid chromatography/mass spectrometry method for SARS-CoV-2 analysis from gargle solutions and saliva. Anal Bioanal Chem.

[19] Dollman NL, Griffin JH, Downard KM (2020). Detection, mapping, and proteotyping of SARS-CoV-2 coronavirus with high resolution mass spectrometry. ACS Infect Dis.

[20] Saadi J, Oueslati S, Bellanger L, Gallais F, Dortet L, Roque-Afonso AM (2021). Quantitative assessment of SARS-CoV-2 virus in nasopharyngeal swabs stored in transport medium by a straightforward LC-MS/MS assay targeting nucleocapsid, membrane, and spike proteins. J Proteome Res.

[21] Rocca MF, Zintgraff JC, Dattero ME, Santos LS, Ledesma M, Vay C (2020). A combined approach of MALDI-TOF mass spectrometry and multivariate analysis as a potential tool for the detection of SARS-CoV-2 virus in nasopharyngeal swabs. J Virol Methods.

[22] Nachtigall FM, Pereira A, Trofymchuk OS, Santos LS (2020). Detection of SARS-CoV-2 in nasal swabs using MALDI-MS. Nat Biotechnol.

[23] Singh P, Chakraborty R, Marwal R, Radhakrishan VS, Bhaskar AK, Vashisht H (2020). A rapid and sensitive method to detect SARS-CoV-2 virus using targeted-mass spectrometry. J Proteins Proteom..

[24] Cardozo KHM, Lebkuchen A, Okai GG, Schuch RA, Viana LG, Olive AN (2020). Establishing a mass spectrometry-based system for rapid detection of SARS-CoV-2 in large clinical sample cohorts. Nat Commun.

[25] Gouveia D, Miotello G, Gallais F, Gaillard J-C, Debroas S, Bellanger L (2020). Proteotyping SARS-CoV-2 virus from nasopharyngeal swabs: a proof-of-concept focused on a 3 min mass spectrometry window. J Proteome Res.

[26] Rivera B, Leyva A, Portela MM, Moratorio G, Moreno P, Durán R (2020). Quantitative proteomic dataset from oro- and naso-pharyngeal swabs used for COVID-19 diagnosis: Detection of viral proteins and host's biological processes altered by the infection. Data Brief.

[27] Ihling C, Tänzler D, Hagemann S, Kehlen A, Hüttelmaier S, Arlt C (2020). Mass spectrometric identification of SARS-CoV-2 proteins from gargle solution samples of COVID-19 patients. J Proteome Res.

[28] Yan L, Yi J, Huang C, Zhang J, Fu S, Li Z (2021). Rapid detection of COVID-19 using MALDI-TOF-based serum peptidome profiling. Anal Chem.

[29] Zuanazzi D, Arts EJ, Jorge PK, Mulyar Y, Gibson R, Xiao Y (2017). Postnatal identification of zika virus peptides from saliva. J Dent Res.

[30] McDonald EE, Goldberg HA, Tabbara N, Mendes FM, Siqueira WL (2011). Histatin 1 resists proteolytic degradation when adsorbed to hydroxyapatite. J Dent Res.

[31] Rabenau HF, Cinatl J, Morgenstern B, Bauer G, Preiser W, Doerr HW (2005). Stability and inactivation of SARS coronavirus. Med Microbiol Immunol.

[32] Sun X, Salih E, Oppenheim FG, Helmerhorst EJ (2009). Kinetics of histatin proteolysis in whole saliva and the effect on bioactive domains with metal-binding, antifungal, and wound-healing properties. FASEB J.

[33] Nair M, Jagadeeshan S, Katselis G, Luan X, Momeni Z, Henao-Romero N (2021). Lipopolysaccharides induce a RAGE-mediated sensitization of sensory neurons and fluid hypersecretion in the upper airways. Sci Rep.

[34] Altschul SF, Gish W, Miller W, Myers EW, Lipman DJ (1990). Basic local alignment search tool. J Mol Biol.

[35] Yoshimoto FK (2020). The proteins of severe acute respiratory syndrome coronavirus-2 (SARS CoV-2 or n-COV19), the cause of COVID-19. Protein J.

[36] Li J, Guo M, Tian X, Wang X, Yang X, Wu P (2021). Virus-host interactome and proteomic survey reveal potential virulence factors influencing SARS-CoV-2 pathogenesis. Med (N Y).

[37] Siqueira WL, Lee YH, Xiao Y, Held K, Wong W (2012). Identification and characterization of histatin 1 salivary complexes by using mass spectrometry. Proteomics.

[38] Nikolaev EN, Indeykina MI, Brzhozovskiy AG, Bugrova AE, Kononikhin AS, Starodubtseva NL (2020). Mass-spectrometric detection of SARS-CoV-2 virus in scrapings of the epithelium of the nasopharynx of infected patients via nucleocapsid N protein. J Proteome Res.

[39] Thomadaki K, Helmerhorst EJ, Tian N, Sun X, Siqueira WL, Walt DR (2011). Whole-saliva proteolysis and its impact on salivary diagnostics. J Dent Res.

[40] Grossegesse M, Hartkopf F, Nitsche A, Schaade L, Doellinger J, Muth T (2020). Perspective on proteomics for virus detection in clinical samples. J Proteome Res.

