# Supplementary material for: Identification of SARS-CoV-2 biomarkers in saliva by transcriptomic and proteomics analysis
Source: Clin Proteomics. 2023 Aug 3;20:30. doi: 10.1186/s12014-023-09417-w (PMC10398966; doi:10.1186/s12014-023-09417-w)
Supplement: Supplementary file 1 — Additional file 1: rRT-PCR amplification curves of stimulated whole saliva samples self-collected by COVID-19 positive individuals using three different collection methods: standard, ice, and RNAlater (Figure S1); SDS-PAGE of stimulated whole saliva samples before and after heat treatment at 60 °C for 30 min (Figure S2). [file 12014_2023_9417_MOESM1_ESM.docx]

**Identification of SARS-CoV-2 biomarkers in saliva by transcriptomic and proteomics analysis**

Lina M. Marin^1^, George S. Katselis^2^, Paulos Chumala^2^, Stephen Sanche^3^, Lucas Julseth^1,2^, Erika Penz^4^, Robert Skomro^4^, Walter L. Siqueira^1*^

^1^ College of Dentistry, University of Saskatchewan, Saskatoon, SK, S7N 5E5, Canada.

^2^ Canadian Centre for Health and Safety in Agriculture, Department of Medicine, College of Medicine, University of Saskatchewan, Saskatoon, SK, S7N 2Z4, Canada.

^3^ Division of Infectious Diseases, Department of Medicine, and Department of Pathology and Laboratory Medicine, College of Medicine, University of Saskatchewan, Saskatoon, SK, S7N 0X8, Canada.

^4^ Division of Respirology, Critical Care and Sleep Medicine, Department of Medicine, College of Medicine, University of Saskatchewan, Saskatoon, SK, S7N 0X8, Canada.

*Correspondence: walter.siqueira@usask.ca; Tel.: +1 306-966-1920

# Additional file 1: Materials and Methods

***Stability of viral RNA in saliva***

Three methods of saliva collection were tested to assess the stability of RNA in saliva samples collected using the SimplOFy™ collection kit (Oasis Diagnostics® Corporation, USA). For that, saliva samples were collected from two male (29.0 ± 7.1 years-old) and two female (23.5 ± 3.5 years-old) individuals diagnosed with COVID-19 by the NPS test done by SHA. Saliva samples were collected on the 5^th^ day after the confirmatory NPS test done by SHA. In the standard method (standard), stimulated whole saliva was collected while holding the collection kit with the hands and placed on a cooler filled with ice immediately after collection. In the ice method (ice), stimulated whole saliva was collected with the collection kit immersed on ice and placed on a cooler filled with ice immediately after collection. In the RNAlater method (RNAlater), stimulated whole saliva was collected while holding the collection kit with the hands, addition of 1 mL of RNAlater (Sigma) upon completion of saliva collection, and placed on a cooler filled with ice immediately after collection. Samples were transported to our research laboratory where SARS-CoV-2 was inactivated at 60°C for 30 min. Viral RNA was extracted from whole saliva supernatant as described in the materials and methods section.

**
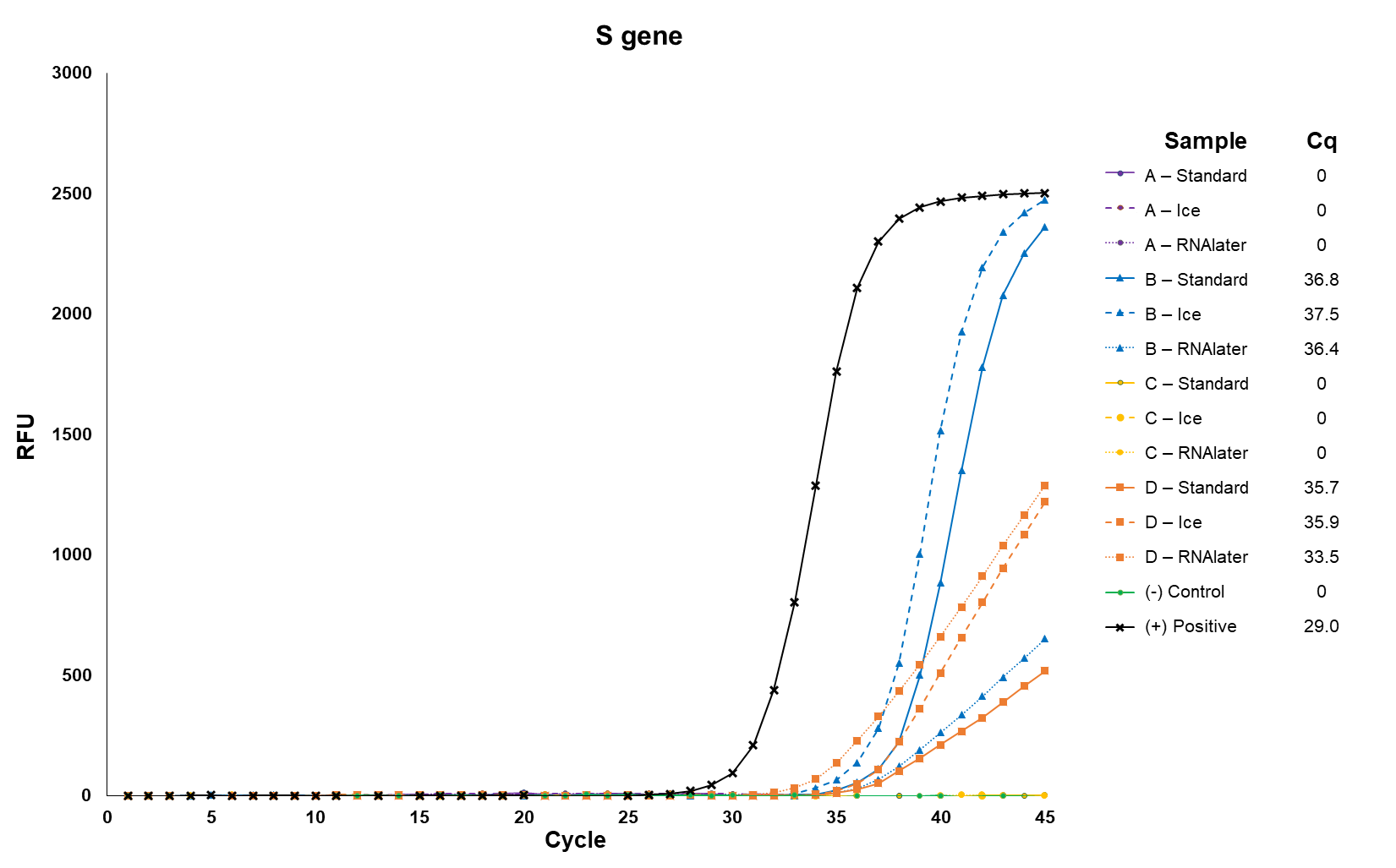
**

Additional file 1: **Figure S1.** rRT-PCR amplification curves of stimulated whole saliva samples self-collected **by four** COVID-19 positive individuals **(A, B, C, D)** using three different collection methods: standard, ice, and RNAlater. Viral RNA (SARS-CoV-2 S gene) was detected in 2 out of 4 samples analyzed (B and D) irrespective of the collection method, with similar Cq values.

***Stability of salivary proteins***

Stimulated whole saliva was self-collected from three healthy volunteers in two different days using a collection kit (SimplOFy™, Oasis Diagnostics® Corporation, USA) without the addition of DNA stabilizers, as described in the materials and methods section. Immediately after collection, saliva samples were placed on ice and then pooled. Two 1-mL aliquots were transferred to two microcentrifuge tubes containing 20 µg of α-amylase 1A (internal standard) each. One aliquot was heat-treated at 60°C for 30 minutes, and the other aliquot was kept on ice to be used as baseline. Whole saliva supernatant was obtained after centrifugation and the total protein concentration was determine by BCA assay (Pierce™, Thermo Fisher Scientific). The equivalent of 20 µg of protein from each sample was dried in SpeedVac (Labconco, Kansas City, MO, USA) and then separated and characterized by sodium dodecyl sulfate polyacrylamide gel electrophoresis (SDS-PAGE). Proteins in the gel were visualized after Coomassie blue staining.

**
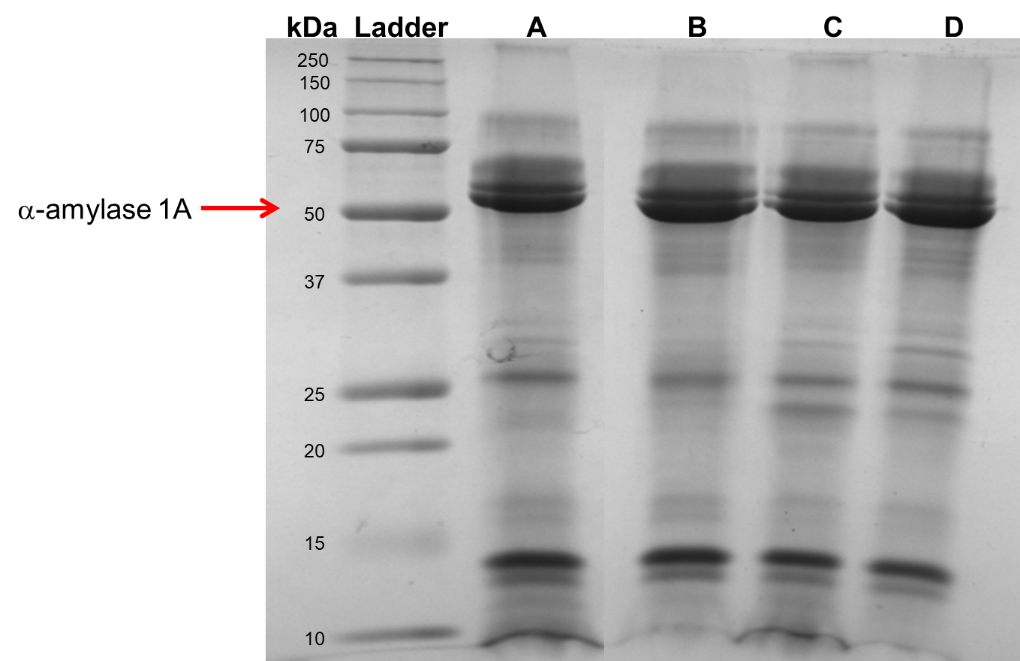
**

Additional file 1: **Figure S2.** SDS-PAGE of stimulated whole saliva samples before and after heat treatment at 60°C for 30 minutes. A) Whole saliva pool day 1, after heat treatment [1,468.1 ug protein/mL]; B) Whole saliva pool day 1, before heat treatment [1,389.7 ug protein/mL]; C) Whole saliva pool day 2, after heat treatment [1,922.6 ug protein/mL]; D) Whole saliva pool day 2, before heat treatment [1,849.1 ug protein/mL]. The band corresponding to α-amylase 1A is indicated by a red arrow. No degradation of proteins was observed after heat treatment.
